# Supplementary material for: Are carbon emissions trading and green financial instruments synergistic? -Comprehensive quantitative research based on content analysis
Source: PLoS One. 2024 Mar 7;19(3):e0298601. doi: 10.1371/journal.pone.0298601 (PMC10919601; doi:10.1371/journal.pone.0298601)
Supplement: S1 Data — (ZIP) [file pone.0298601.s001.zip › Data/地级市Z_F工作报告.zip!/║┌┴·╜¡/╞δ╞δ╣■╢√╩╨/└┤╫╘╣1⁄2╓┌║┼í╛┬φ┐╦╩2╛▌═°í┐_2008.docx]

各位代表：

　　现在，我代表市政府向大会报告工作，请予审议，并请市政协各位委员提出意见。

　　一、2007年工作回顾

　　2007年，我们圆满完成了市十四届人大一次会议确定的各项工作任务，国民经济和社会发展取得显著成绩。全市地区生产总值预计完成564.5亿元，增长15%，是改革开放以来发展最快的一年。财政总收入完成46亿元，为预算的130.8%，增长26.9%，地方财政收入完成28亿元，为预算的146.2%，增长28.9%；财政总支出完成101.2亿元（包括上级财政补助支出），增长29.3%，地方财政支出完成71.6亿元，为预算的122.4%，增长28%。城镇固定资产投资完成159.1亿元，增长31%。

　　一年来的主要工作是：

　　（一）加快调整经济结构，不断提高运行质量，国民经济持续快速增长。通过抢抓国家振兴东北老工业基地的历史性机遇，在努力破解制约国企改革发展诸多难题的基础上，开始实施大企业翻番、民营企业跨越、优势产业集群化发展“三大战略”，推进了工业经济科学跨越发展。规模以上工业完成增加值138.2亿元，增长34.7%，预计完成利润23亿元、税金24亿元，分别增长90.1%和45%，经济效益综合指数预计达到155，提高了30个点。万元GDP综合能耗降低4%。重点骨干企业发展势头良好，有11户企业跻身省87户重点工业企业行列，8户企业主营业务收入跻身全省50强。民营经济快速增长，占国民经济比重达到41.1％；优势产业集聚度进一步提高，装备制造业和食品加工业增加值占全市规模以上工业增加值的75.1%，对经济的拉动作用进一步增强。哈大齐工业走廊齐齐哈尔规划区建设取得新进展，依安、富裕、铁锋园区和一重集团列入哈大齐工业走廊总体规划；近三年来累计入区企业107户，总投资58亿元，其中2007年新开工建设项目20个，投资10.7亿元。富拉尔基开发区被列为省中小企业创业基地，累计入驻企业24户，实现技工贸收入8.2亿元。扎实推进新农村建设，农村经济全面发展。努力克服严重干旱等自然灾害，粮豆薯总产107.6亿斤。畜牧业产值实现80亿元，增长4.5%，占农业总产值的38%。绿色食品、有机食品和无公害农产品认证数量分别增长7.1%、67%和1.5倍，主要农作物全部实现了无害化种植。以抓企业上项目为突破口，推进县域经济发展，财政收入超亿元的县达到7个。农业产业化水平明显提高，龙头企业达到155家，农村合作经济组织健康发展，十三条产业链发展较快，带动了农业的标准化、规模化，促进了农区工业化。投入农田水利工程建设资金2亿元，增长37.4%，新增农机作业合作社56个，改善了农业生产条件。累计转移农村劳动力90万人，占农村劳动力总数的51.9%，实现劳务收入40.5亿元，增长25%。落实支农惠农政策，发放综合直补、粮食、生猪、奶业、良种等补贴资金10亿元。为新农村建设试点村投入帮建资金4856万元。163个贫困村实现整体脱贫，农村贫困人口下降了8.3%。现代服务业发展较快，第三产业不断壮大。加大了旅游品牌推介力度，包括我市在内的“情系黑土地”旅游线路荣获中国十大完美旅游线路之首。实现旅游收入33亿元，创汇470万美元，分别增长12.1%和26.7%。会展业加快发展，市场化运作水平有所提高，成功举办了绿博会、关东文化旅游节、小商品交易会、重装会以及汽车工业博览会、华夏丝绸服装博览会等展会。金融环境有所改善，各项贷款余额纯增21亿元。物流业发展势头良好，“万村千乡”市场工程基本覆盖全市乡村。社会消费品零售总额实现210.5亿元，增长17.1%。

　　（二）深化各项改革，扩大对外开放，经济社会发展活力明显增强。国企改革攻坚进入收尾阶段，在改制成本严重不足的情况下，完成了7户困难企业的产权制度改革，化解债务2.3亿元，盘活资产5946万元。10户企业列入国家政策性破产计划。中直企业办社会职能全部分离，一重集团公司厂办大集体改革开始试点。整合齐重数控公司和齐二机床集团铸造资源，建设铸锻造基地，成立了腾翔重型铸锻造有限责任公司。克服重重困难，黑龙股份债务重组基本结束，可化解历史债务34亿元，为盘活存量资产、如期恢复上市奠定了基础。经过积极争取和协调，整合了原“齐二药”资源，为新企业生产创造了条件。下大决心，花大气力，解决了多年积累的大田集团问题。农村综合改革全面铺开，林权制度改革试点顺利进行。市级312个预算单位已纳入国库集中支付范围，实行了公务接待费货币化管理、公务人员出差定点采购、市级离退休领导干部用车费用定额包干等制度，探索了规范职务消费新机制。行政审批制度、招投标制度、社会保障制度、事业单位产权制度等改革取得新成效。进一步规范了出租车市场管理。招商引资取得新成果，直接利用外资4885万美元，增长21.9%。组织参加国内外重大招商活动17次，世界500强企业英糖公司以及香港国际生态安全管理集团、康师傅集团、北京国华风力发电公司、蒙牛集团等一批国内外知名企业落户我市。沃尔玛超市开业，新玛特休闲购物广场、甘南飞鹤乳业等项目进展顺利。与80多个国家和地区建立了贸易联系，对俄经贸科技合作领域进一步拓宽。进出口总额预计完成2.6亿美元，增长81%。与乌克兰马里乌波尔市和国内的盐城市、呼伦贝尔市、泸州市结为友好城市。齐齐哈尔--北京南苑新航班开航。国家和省7个大型专业会议相继在我市召开，接待服务水平进一步提高。

　　（三）注重统筹发展，加强基础设施和生态环境建设，城乡面貌有新改观。建设投资重点向农村转移，城乡建设总投资完成38.1亿元，其中村镇建设和农村道路建设投资21.1亿元。建设农村公路3752公里，其中通乡通村公路2718公里。新建了10处农村客运站。市区新建和改造道路16条，铺装面积25.1万平方米。新安装和升级路灯1060盏。完成了北出城口道路两侧拆迁和管线施工任务。中心城区热电联产热网A区一期工程建设如期竣工，2.2万户、171万平方米热负荷并网供热，每年节约标煤2.3万吨，减少烟尘943吨、二氧化碳667吨。大庆至昂昂溪的天然气输气管道工程基本完工。第二医院综合楼投入使用，医学院主体工程建设如期完工。新建住宅70.8万平方米，市区人均建筑面积22.6平方米。清理整顿违章建筑1811处。111国道齐齐哈尔至讷河段全线通车。努力改善生态环境，完成人工造林25.8万亩，森林覆盖率提高到13.3%。对中心城区22条主次干道和9个交叉路口进行了新植、密植、补植绿化，城区绿化覆盖率达到30.8%。实施了明月岛、鹤泉园升级改造和劳动湖三期治理工程。化学需氧量和二氧化硫排放量有所下降，城市空气质量二级以上天数达到327天。

　　（四）落实科教兴市和人才强市战略，不断加大投入，社会事业全面发展。加快创新型城市建设，科技支撑能力不断增强。新增民营科技企业85户，总户数达到917户。高新技术企业实现产值103.6亿元、利税10.1亿元，分别增长46.8%和25.9%。实施市级科技项目105项，取得科技成果104项。有51个项目获市级科学技术奖，一重集团公司的1.5万吨锻造水压机荣获省科技进步唯一特等奖，齐轨道交通装备公司的K2型转向架获一等奖。成立了市科技企业孵化中心，已有17家企业入驻。强化知识产权保护意识，申报专利补贴项目67项。人才队伍建设得到加强，形成市级重点学科带头人梯队84个，31名专家分别享受国务院、省政府和市政府特殊津贴，28名科技有功人员获市长特别奖。人才交流日益活跃，举办了96场次招聘活动，提供就业岗位8.6万个，市人才市场已成为人力资源配置的品牌阵地。教育事业有新发展，促进了教育公平。“两基”覆盖率、农村中小学信息化设备覆盖率均达100%，远程教育工程建设全省领先。创造性开展学校艺术体育教学，素质教育取得新成果，教育教学质量进一步提高。落实“两免一补”资金1.5亿元，88万人次农村中小学生受益。停止了8所热点学校公有民办招生，全面恢复了公办性质。职业学校招生2.15万人，占高中阶段教育招生的47%，毕业生就业率达95％。积极探索农村综合中学农科教相结合办学模式，提高了为新农村建设的服务能力。齐齐哈尔大学、医学院、职业学院顺利通过国家教育部教学工作水平评估和验收。文化和体育事业取得新的成果。群众性文化活动位居全省前列。文化阵地建设得到加强，新建6个乡镇文化站，市图书馆新馆开工建设。我市策划编排的第七届绿博会大型文艺演出、首届和平节、龙沙公园百年庆典等活动圆满成功。全市有6个项目入选省级非物质文化遗产名录，金东北路界壕边堡被列入全国长城资源调查系列，金长城与世界文化遗产长城系列顺利接轨。承办了2007年甲级A组世界冰球锦标赛，首开在亚洲举办国际甲级冰球赛事的先河。我市首次组团参加全国城市运动会，金牌总数和积分获得东北地区第二名。第十一届全国冬运会各项准备工作基本就绪。医疗卫生服务体系进一步完善。投资1574万元，新建乡镇卫生院23个，改善了农村医疗服务环境。社区卫生服务中心和卫生服务站发展到63个，覆盖人口85%以上。实施了“生殖健康促进计划”，我市的生殖健康援助工作经验在全国推广。荣获全国“双拥模范城”五连冠。

　　（五）千方百计办好大事实事，高度重视并切实改善民生，人民生活水平得到提高。城镇居民人均可支配收入达到9561.7元，农村居民人均纯收入达到2752元，分别增长19.5%和6%。与人民群众生产生活密切相关的41件大事实事基本完成。深入实施创业带就业工程，在2006年为10743名下岗失业人员发放小额担保贷款21100万元、鼓励和带动48200名下岗失业人员实现了创业和稳定就业的基础上，2007年又为11000名下岗职工发放小额担保贷款21753万元，在全国百家重点城市排序中继续保持第五位。开展各类职业技能培训5.8万人次。为13.5万人发放社会保险补贴1.66亿元。为零就业家庭安排3000个公益岗位，在全省率先实现了零就业家庭动态为零的目标。城镇新增就业11.6万人，失业率控制在4.2%以内。完善社会保障体系，保障能力显著增强。全市养老保险费收入完成12.47亿元，养老金支出15.66亿元，保证了按时足额发放。超额完成了失业保险基本征缴目标。初步建立了覆盖城乡居民的医疗保险制度。新型农村合作医疗参合农民238万人，参合率达到91%，提前3年实现了基本覆盖农村居民的目标；积极争得国家城镇居民基本医疗保险试点，自去年11月份全面启动以来，已有10.25万城镇居民参保；城镇职工基本医疗保险参保人数达82万人。最低生活保障制度由城市向农村延伸，为城市15.8万低保对象发放低保金1.36亿元，为农村11.3万低保对象发放低保金3400万元，发放各类救助资金1亿元，实现了应保尽保。为72万农民下拨救灾资金3144万元，使农村灾民得到及时救助。建设安居工程6.1万平方米，解决了960户低收入居民住房问题。实施残疾人康复和扶贫工程，免费为400名贫困残疾白内障患者实施了复明手术，帮助600户贫困残疾人家庭新建或改建了住房。完成了北华等小区的47栋楼、3245户居民的天然气管道安装，新建、翻建、维修了中心城区204座公厕。提高了社区工作人员待遇，社区服务功能得到加强。

　　（六）加强民主法制建设，坚持依法行政，经济发展环境不断改善。制定出台了市政府重大决策规则，科学民主决策程序进一步完善。及时向市人大报告工作、向市政协通报情况，办理人大代表议案102件、政协委员提案279件，办结率均达100％。起草、审修地方性法规（草案）3部、政府规章（草稿）12件，重新梳理确认了行政机关的执法依据和执法权限。调整规范了市政务服务中心审批流程和时限，提高了服务质量，全年共受理各类服务事项11万件，办结率达到99.9%。加大了警示教育和案件查处力度，强化了社会监督和舆论监督，廉政建设进一步加强。新闻媒体的“市长与市民”、“纠风直通车”、“纠风热线”、“科所队走进直播间”等节目受到市民好评，行风评议活动取得实效。启动了“诚信鹤城”建设工作。安全生产形势总体稳定，生产事故下降50.7%。认真贯彻落实《信访条例》，积极处理新形势下的人民群众来信来访，解决了一批积案和遗留问题。深入推进“平安鹤城”建设，社会治安综合治理成效显著，人民群众的安全感明显增强。

　　民族宗教、外事侨务、机关事务管理、审计、统计、档案、地方志、人防、气象、妇女儿童、老龄、民兵预备役等工作取得了新成绩。

　　各位代表，过去的一年，是我市抢抓机遇、破解难题、经济社会又好又快发展的一年，是增加投入、奠定基础、积攒发展后劲的一年，是以人为本、改善民生、让人民群众共享改革发展成果的一年，是政治稳定、社会和谐、凝心聚力、奋发进取的一年。一年来所取得的成绩，是省委、省政府和市委正确领导的结果，是市人大、市政协监督支持的结果，是全市人民共同奋斗的结果。在此，我代表市人民政府，向全市各族人民、各民主党派、各人民团体和各界人士，向所有关心、支持我市改革发展的同志们、朋友们，表示衷心的感谢并致以崇高的敬意！

　　我市经济社会发展虽然取得了显著成绩，但用科学发展观和人民群众的期盼来衡量，还存在一定的差距和问题，有些方面的差距和问题还比较突出和明显。主要是，企业自主创新能力总体不强，发展方式相对粗放，集群化水平低，经济效益不高，资金、运输等瓶颈问题依然突出；农业基础设施薄弱，产业化程度较低，抵御自然和市场风险的能力脆弱，农民增收的渠道不宽、基础不牢。在和谐社会建设方面还存在一些薄弱环节，对发展社会事业的投入不足，社会利益诉求机制不完善，劳动就业、社会保障、教育、医疗、居民住房等关系群众切身利益的问题还比较多，特别是一些生活必需品和生产资料价格上涨较快，给人民群众尤其是弱势群体生产生活带来一定的影响。政府职能转变不到位，一些地方和部门思想不够解放，抢抓机遇意识不强，缺乏创新性思路和突破性措施，部门、行业不正之风和腐败现象还不同程度地存在。对于这些问题，我们在今后的工作中，一定要采取有针对性的措施，认真加以解决。

　　二、2008年工作任务2008年是全面贯彻落实党的十七大精神的第一年，是实施“十一五”规划承上启下和加快老工业基地振兴的关键一年，也是改革开放30周年，做好今年的工作，意义重大，至关重要。

　　今年的政府工作要以党的十七大精神和中国特色社会主义理论体系为指导，认真贯彻落实市委十一届三次全会精神，进一步解放思想，深化改革开放，强化一产优势，提升二产竞争力，做大三产规模，推进产业结构优化升级，着力改善民生，促进社会和谐，实现经济社会又好又快发展。

　　综合考虑各种因素，今年全市经济社会发展的主要预期目标为：地区生产总值增长10%以上，地方财政收入增长12%，城镇固定资产投资增长15%，规模以上工业增加值增长25%，社会消费品零售总额增长9%，进出口总额增长20%，城镇居民人均可支配收入增长8%，农村居民人均纯收入增长5%，居民消费价格总水平涨幅控制在4.5%左右，新增就业10万人，城镇登记失业率控制在4.6%以内，人口自然增长率控制在3.04‰以内，万元GDP综合能耗下降6%以上。

　　重点做好以下几项工作：

　　（一）深入实施“三大战略”，推进工业经济科学跨越发展。把工业作为加快经济发展的首要任务、财政收入的主要来源、促进就业的重要载体来抓，通过实施“三大战略”，转变发展方式，走现代工业、现代农业、现代服务业全面发展的新型工业化道路，使工业经济尽快跃上结构优、质量好、总量大、竞争力强的新台阶。提高企业自主创新能力。充分发挥市场机制的促进作用和政府扶持的导向作用，支持创新要素加速向重点企业集聚，促进“政、产、学、研、金、介”相结合，加快研发拥有自主知识产权的核心技术，推动骨干企业技术改造。实施品牌战略，鼓励和支持企业争创“中国名牌产品”、“中国驰名商标”、“出口名牌产品”。滚动实施技术创新项目120项，重点搞好20个以上具有自主知识产权的新产品开发。提高信息化水平，用信息技术提升企业发展能力。加速大企业翻番。推动大企业战略重组，支持一重集团公司、齐轨道交通装备公司、齐重数控公司、齐二机床集团等大企业加快发展。促进工业企业梯次增长，力争3户中型企业晋升为大型企业，3户小企业晋升为中型企业。飞鹤、浩盈、北疆、北大仓等企业力争进入全省50强。加快工业大项目建设，实施总投资5000万元以上项目46个，其中超亿元项目24个，年内竣工投产11个。重点推动一重铸锻钢基地及大型铸锻件项目、齐重数控及齐二机床高档重型数控机床产业化项目、北京安邦石化有限公司齐齐哈尔分公司30万吨烧碱、30万吨聚氯乙烯、66万吨水泥熟料生产、齐纸恢复生产和技术改造等一批大项目建设。各县（市）重点建设一两个富有竞争力、支撑力、牵引力的大项目。促进民营企业跨越发展。把促进民营企业发展作为各级政府的重要职责，大力推动民营经济上层次、扩规模、增效益，发展壮大一批立区、立县、立市的民营企业。实施扶优扶强战略，鼓励并支持民营骨干企业做大做强。加强培训服务，壮大职业经理人和专业技术人才队伍。支持建立各类行业协会，规范民营企业发展。推动优势产业集群化发展。以建设国内重型装备技术研发中心、装备工业基地和绿色食品产业基地为目标，鼓励和扶持两大优势产业延伸壮大产业链条，提升产业集聚度。加快大企业裂变和配套产品扩散，以富拉尔基开发区、龙沙区、铁锋区和昂昂溪区的闲置资产为主要载体，着重发展配套件加工和铸锻造产品，通过政策扶持等措施，吸引市内外企业和民间资本进入园区发展配套加工业。扶持腾翔重型铸锻造有限公司加快发展，增强协作配套能力。创造性地落实支持企业发展的政策措施。深入研究《东北地区振兴规划》和国家的各项支持政策，最大限度地争取和落实好政策。不折不扣地落实我市鼓励企业发展的税收支持政策、财政扶持政策和政府奖励政策。实行大中型企业纳税、发展规模和投资增长年度排名制度，每年两次在新闻媒体发布。坚持各级领导联系包扶重点企业制度、部门帮办责任制度，严格执行改善经济发展环境的工作制度和责任追究制度。积极搭建有利于企业发展合作的配套协作平台、融资平台、宣传推介平台和信息服务平台。支持各家商业银行开展金融创新，积极向上争取贷款规模，发挥好担保资金作用，加大为企业融资力度。积极推进“诚信鹤城”建设，完善企业和个人征信制度，建设银企共赢的金融生态环境。加大节能减排工作力度。落实节能减排国策，严格实施《节能减排综合性工作方案》，加强对节能减排的统计、监测和管理，加快淘汰高耗能、高污染行业的落后产能、工艺和设备。全面推行排污许可证制度，严把新开工项目环评关，将主要能耗环保指标坚决控制在国家规定的标准内。做好全国污染源普查工作。在重点行业和重点企业开展节能降耗对标达标活动，严格煤电机组脱硫设施建设标准，加快电力和化工等企业的脱硫设施改造。如期完成水泥产业布局调整。积极向上申报污染治理项目，争取国家治理项目投资，推进技术含量高、资源消耗低、环境污染少的项目建设。搞好新型墙材的研发和推广，实现2008年底中心城区“禁实”的目标。积极争取国家资金和技术支持，加大旧楼节能保温改造力度。开发和推广节约、替代、循环利用和治理污染的先进适用技术，大力发展循环经济，继续抓好富拉尔基区循环经济试点工作。全面开工建设列入松花江流域治理的11个项目。完善和落实节能减排政策措施，将节能减排作为政绩考核的重要指标，实行严格的问责制和一票否决制，增强全社会的节能减排意识，建设资源节约型、环境友好型社会。

　　（二）大力发展现代农业，加快社会主义新农村建设。抓住国家大幅度增加对农业农村投入的机遇，积极争取并认真落实各项强农惠农政策，深入推进县域经济“四个换位”，加强农业基础设施建设，大力发展农村社会事业，促进城乡经济社会一体化发展。着力提升农业产业化水平。从延伸产业链入手，打破区域界限，突出抓好加工企业、农村合作经济组织、农贸市场和原料基地建设，建立企业与基地之间、县域之间紧密型的利益联结机制，构建布局合理、主导产业特色突出、组织化程度高的新型产业化格局。通过争取国家政策支持、引进知名企业、改造现有企业等措施，做大做强龙头企业。积极引导和支持有条件的农民专业协会向专业合作社转变，引导各类市场主体参与农业产业化经营，健全农村市场体系，培育农村流通主体，大力推进农村信息化，为农业产业化发展提供有效服务。努力提高粮食综合生产能力。充分利用国家加大对粮食主产区的扶持力度、加大对保护和提高农业综合生产能力的补贴力度、加大对农民的直接补贴力度的政策机遇，突出绿色和特色产品生产，科学调整作物区域布局，加强农业科技开发与推广，努力扩大优质粮食生产基地规模。重点建设好800万亩优质玉米生产区、500万亩高产高油高蛋白大豆生产区、180万亩马铃薯生产区和200万亩水稻生产区，形成2000多万亩优质粮食作物产业带。大力推广标准化种植，绿色作物面积达到770万亩。大力发展畜牧业生产。优化畜牧业养殖结构和布局，推进规模化、标准化养殖，建设优质畜牧业基地。利用国家扶持政策，突出抓好生猪生产和奶源建设。加强动物疫病防控，抓好无规定疫病区建设，实施优质安全工程，强化乡村畜牧综合服务基础设施建设，健全畜牧产业保障体系。畜牧业产值达到83亿元。积极发展农村劳务经济。搞好阳光工程培训，推进劳务经济市场化运作，加强劳务服务体系建设，鼓励和引导农村劳动力向国外、埠外、小城镇和非农产业转移，力争累计转移93万人，实现劳务收入46亿元。加强农业基础设施建设。以水利设施、治理沙化、改良土壤、改善生态为重点，谋划和包装好农业基础建设项目，积极争取国家各类专项资金支持，重点加大对龙甘泰等西部旱区和农业基础薄弱地区的投入。新打抗旱水源井3530眼，新增节水喷灌面积10万亩，新增和改善除涝面积10万亩，水土流失治理面积45万亩，扩大移动式滴灌和保护性耕作面积。实施“沃土工程”，抓好黑土流失和土地沙化治理。大力发展农机合作组织，组建农机作业合作社50个，综合机械化程度达到82%。加强生态屏障建设，造林20万亩，种草10万亩，草原改良治理“三化”10万亩。扎实推进新农村建设试点工作。加快培育试点村的主导产业，推进“一村一品”工程。突出抓好薄弱试点村建设，同步完成试点任务。继续组织社会力量做好帮建工作。

　　（三）突出发展以现代服务业为重点的第三产业，增强对经济增长的拉动作用。把第三产业作为财源建设新的增长点，积极培育，壮大规模，提高比重，加快发展。大力发展旅游业。继续推进九大系列旅游产品建设，重点开发集生态人文与养生休闲于一体的避暑旅游、冰雪旅游，积极发展滨江度假、乡村度假等旅游精品，高水平开发温泉地热项目。加强旅游基础设施建设，大力开发建设扎龙、明月岛、尼尔基斯湖等主要景区，丰富旅游景区文化内涵。加大旅游商品研发生产力度，延伸旅游产业链。加强与我省西部城市及呼伦贝尔市的区域旅游合作，共建东北大旅游格局，实现客源与资源共享、人流与资金流互通。制定优惠政策，优化旅游业发展环境。积极发展会展业。制定促进会展业发展的优惠政策，积极引进和培育壮大专业会展公司，对引进展会有功人员按招商引资政策兑现奖励，设立会展业发展资金。高水平办好绿博会、小交会、关东文化旅游节，发展品牌展会，培育综合展会和专业展会。支持会展中心发展，完善会展中心功能，增强招展办展能力。成立会展工作机构，加强行业管理，统筹会展业发展。培育现代物流业、服务外包业和中介服务业。规范物流秩序，整合物流资源，加快物流业发展。引导工商企业物流社会化、物流企业经营专业化，促进装备制造业、绿色食品产业与物流业有机融合、互动发展。发挥市道路货物运输结算中心作用，支持北大仓物流中心建设专业化物流园区，打造区域性物流基地。积极落实服务外包企业发展规划，建设装备制造业外包产业园区。鼓励创办技术研发、市场开拓、营销代理、售后服务等经济实体。大力发展融合现代信息和高新技术的新兴服务业，加快电子商务、信息咨询等新型业态发展。规范发展会计、法律、房地产、资产评估等中介服务业。完善城乡市场体系建设。加强城乡市场消费服务网络建设，促进消费结构升级。加快发展面向城乡居民的生活性服务业，推动商贸连锁、物业管理、公共服务等发展。继续支持社区服务业发展，推动便民消费服务进社区、进家庭，发展一批社区商业示范区。完善“万村千乡”市场工程。

　　（四）加快城乡建设，改善人居环境。从今年起，开展创建国家园林城市、国家环保模范城市、国家卫生城市、国家森林城市、国家节水型城市的“五城同创”工作，进一步提升城乡建设整体水平，创造良好的人居环境和经济发展环境，提高城市知名度、影响力、竞争力。按照“五城同创”各项指标要求，坚持“四高”原则，统筹规划，合力推进，力争在5年内实现既定目标。强化城乡建设规划。发挥规划的龙头作用，结合“五城同创”目标，高起点搞好城乡规划。启动编制沿江绿化带和大城区绿化规划，启动江西开发区项目建设防洪规划，超前完成旧城区改造、近期城市发展规划，完成全市乡镇建设规划。加强城乡规划一体化管理，实行规划设计招投标。加强土地使用规划，完善土地储备和招拍挂制度。严格执行城乡建设规划。改善城乡生态环境。重视人与自然协调发展和人居环境建设。突出抓好龙华路、卜奎大街、站前广场、南苑转盘广场绿化升级改造。市区植树30万株，栽花、铺草、植绿篱100万平方米。继续搞好绿色学校、绿色企业、绿色小区建设。改善中心城区环卫设施，提高城市生活垃圾无害化处理率，防止城市污染向农村转移。加强环城防护林建设和湿地保护。推进乡村清洁工程，改善农村人居环境。加强环境监测监管，严防噪声、废物、废液、废气、危险化学品等污染环境。加快城乡基础设施建设。完成中心城区热电联产热网A区二期工程建设和小锅炉房整合撤并。加快天然气复线工程建设，实现昂昂溪至富拉尔基和至中心城区两条支线建成投产。加大二次加压供水的改造和整合工作力度，年内完成77座泵站的改造与整合。完成劳动湖三期综合治理工程及北出城口路、水师路、西园街等主次干道建设。搞好中华路、龙华路、新明大街、中心广场、站前广场等街路广场装饰的亮化美化。加强房地产市场调控，调整商品房供应比例，扩大棚户区改造面积。完善廉租住房保障制度，加快保障性住房建设，对21724户“双困”户实行廉租补贴。推进小城镇、中心村、试点村、城中村建设。加强和完善城乡公路网络建设，积极启动齐富高等级公路建设，配合搞好齐大高速公路，111国道齐市至泰来段、讷河至嫩江段，鸡讷公路鸡西至拜泉段建设。新建通乡通村公路1500公里。支持抓好哈大齐城际铁路、齐齐哈尔大件码头等重点工程建设。做大做强市城投公司，增强资本运营能力，多渠道筹措城市建设资金。加大城乡管理力度。完善城市管理体制，制定实施城乡违法建筑查处办法，探索长效管理机制。继续实行“门前三包”，促进城市社会化管理。推进城乡管理由主干道向次干道、由商业区向居民区、由县级镇向建制镇延伸。加大对牌匾条幅、乱贴乱画等立面容貌的整治力度，规范市场秩序、交通秩序。认真贯彻《物权法》，依法规范物业公司权利和义务，切实保护业主利益。加快中心城区地理信息公众服务平台建设，促进城市管理数字化。

　　（五）坚定不移地深化各项改革，在重点领域和关键环节取得突破。把改革创新精神贯彻到工作的各个环节，紧紧抓住重要战略机遇期，着力突破体制障碍，提高改革决策的科学性，增强改革措施的协调性，不断改善经济社会发展的制度环境。深化国有企业改革。研究制定扶持政策，支持一重集团公司、齐二机床集团、齐轨道交通装备公司、东府塑料、华工机床、东兴农机、运建集团等企业上市融资，抓好黑龙股份恢复上市工作。加快国有企业产权制度改革，争取尽快终结已进入司法程序的造纸厂、磷肥厂、油脂化学厂等10户企业的政策性破产。健全法人治理结构，完善现代企业制度。加强国有资产监管，探索建立国有资本预算制度。继续分离企业办社会职能，做好地方接收工作。按照国家和省政府的部署，认真解决好企业工资历史拖欠问题。深化农村改革。积极推进农村综合改革，重点抓好土地流转、农机合作社、场县共建、农民合作经济组织、化解乡村债务、为农服务代理制等项改革。创新农业科技推广体系，加快农业标准化推广进程。切实抓好林权制度改革试点工作，力争在今年4月末前完成集体林权制度改革试点，并逐步在全市推开。推进畜牧兽医管理体制改革，为畜牧业发展提供制度保障。深化财政金融体制改革。培育壮大财源项目，积极争取财源建设资金。加强县级财源建设，各县的财政收入力争全部超亿元，部分县达到2亿元。完善公共财政体系，优化财政支出结构，实行县（市）区国库集中收付制度。完善政府采购制度，创新政府采购模式。鼓励和支持地方性商业银行做大做强。深化农村金融体制改革，发育多种形式的农村金融机构，创新农村金融服务方式，大力发展小额信贷业务，努力解决农民贷款难问题。探索建立政策性和商业性相结合的农业保险制度，降低农业生产风险。深化社会事业体制改革。完善城乡义务教育经费保障机制，认真落实国家免收城市中小学生学杂费政策。按照国家和省的统一部署，积极稳妥地推进医药卫生体制改革。坚持公共医疗卫生的公益性质，适时实行政事、管办、医药、营利性非营利性分开，创新公立医疗卫生机构的体制和服务机制。整合卫生资源，开展市县乡医疗机构联合经营。推进以人事、分配制度为重点的文化事业单位改革，支持非公经济发展文化产业，加快演艺机构集团化重组。深化行政管理体制改革。按照国家和省的统一部署，积极推进行政管理体制改革，优化政府机构设置，创新行政管理机制。同步推进社会保障、事业单位、户籍制度等领域的改革。

　　（六）进一步扩大对外开放，加快发展开放型经济。坚持引进来与走出去相结合，努力提升发展的外向度，以大开放促进大发展。进一步做好招商引资工作。坚持专业化、市场化、园区化、产业化招商引资的路子，突出引进战略投资者和大项目。优化招商引资项目库，增强项目投资吸引力。进一步加强同长三角、珠三角、环渤海、港澳台等重点地区的联系，推进与友好市、区的务实合作。进一步完善服务设施，高标准做好接待服务工作。提高园区建设水平。积极破解土地利用难题，解决个别园区企业长期占地不建设的问题。鼓励多渠道筹措资金，增加对园区和产业基地的投入，加强基础设施建设。列入哈大齐工业走廊建设的园区努力达到“七通一平”的要求，引进植入更多更好更大的项目，使其成为产业聚集的重要基地和高新技术产业的孵化中心。完善服务机制，为入驻企业提供高效便捷的服务。加强对外经贸工作。扩大对俄经贸合作，推动对韩贸易升级。加快出口企业产品结构调整，扩大装备技术产品出口；提高农产品品质，增强出口产品竞争力。创新对外投资合作方式，鼓励优势企业到境外开展能源原材料投资合作、承接工程和项目，带动优势产品出口和劳务输出。加强同大连、满洲里、黑河等口岸城市的合作，积极探索通畅便捷、互惠共赢的贸易机制。

　　（七）以解决好人民群众最关心、最直接、最现实的利益问题为重点，着力保障和改善民生。认真组织实施省委、省政府确定的“十大惠民工程”，立足当前，着眼长远，坚持从办得到的事情做起，继续抓好大事实事，千方百计解决人民群众关心的热点、难点问题。通盘考虑、分类指导城乡就业和劳动力转移工作。大力实施创业带就业工程。全面落实《就业促进法》和各项就业政策，不断拓宽就业渠道，扩大就业容量。加大小额担保贷款工作力度，积极引导和鼓励劳动者自主创业、灵活就业。积极开发公益性岗位，完善面向所有弱势群体的就业援助制度，帮助零就业家庭解决就业困难。依法加强劳动监察，保障劳动者合法权益。加强就业培训，建设统一规范的城乡劳动力市场，发展就业中介服务组织，创造良好的就业环境。加快社会保障体系建设。完善城乡最低生活保障制度，不断提高城乡低保水平。继续扩大养老保险覆盖面，提高退休职工待遇，确保春节前养老金调整发放到位。探索建立农村养老保险制度。积极做好城镇居民基本医疗保险试点工作，扩大参保面，将从事建筑业的外来农民工纳入工伤保险范围。做好贫困人口医疗救助工作，建立健全法律援助体系，扩大援助覆盖面。发展社会福利和慈善事业。从人口老龄化的实际出发，加快发展老龄事业和老龄产业。加强“五保”供养工作，落实新的集中和分散供养标准，加快敬老院等“五保”供养的基础设施建设。做好扶贫解困工作。积极帮扶弱势群体解决生产生活困难，继续开展“一帮一”和“对口支援”等扶贫活动，确保帮扶对象基本生活。以贫困户增收为核心，加强重点村中心屯基础设施建设和公益事业建设。加大对革命老区贫困村的扶持力度。增强基层组织的服务功能。搞好第二届社区换届选举，开展业务培训，提高社区工作人员服务意识和能力。开展和谐社区创建活动，完善社区服务体系，拓宽服务领域和渠道。推进农村社区建设试点。健全和完善村民自治制度，开展村务公开民主管理示范单位创建活动，促进民主决策和管理。维护社会稳定。强化安全生产管理和监督，认真落实安全生产责任制，加强生产、交通、防火安全隐患的排查和治理，坚决杜绝重特大事故发生。继续开展食品安全和产品质量专项整治活动，依法规范食品、药品、饮用水等生产经营行为，确保公共安全，切实维护群众利益。深入开展“平安鹤城”创建活动，搞好社会治安综合治理，完善城乡打防控一体化体系，严厉打击各种犯罪行为，增强人民群众的安全感。认真处理人民群众来信来访，坚持领导接访、以情处访、依法治访，落实信访工作责任制和领导干部信访接待日、重点案件包案制等制度，努力化解信访矛盾和问题。完善突发事件和群体性事件应急管理机制。开展“五五”普法教育，加快依法治市进程。认真落实国家价格补贴政策，加强市场和价格监管，严厉打击哄抬物价行为。

　　（八）大力发展社会事业，实现经济社会全面进步。更加注重社会建设，扩大公共服务，完善社会管理，促进经济社会协调发展。优先发展教育事业。推进素质教育，优化教育结构，提高教育现代化水平，全力办好人民满意的教育。优化教育资源配置，加快普及高中阶段教育。不断创新职业教育形式，全面提高农村综合中学和县级职教中心建设水平。重视学前教育，关心特殊教育。加大校长和农村教师培训力度，提高校长和教师整体素质。整合院校品牌资源，提高高等教育质量。鼓励和规范社会力量兴办教育，发展远程教育和继续教育。深入实施人才强市战略，围绕提高自主创新能力，关心和重视本地人才，引进和开发适用人才，逐步形成总量科学合理、人才结构与经济结构基本适应、整体素质明显提高的人才队伍。完善符合各类人才特点、促进人才全面发展的用人、分配和评价制度。发挥市场配置人才资源的作用，加强人事公共服务体系建设，提高人才对经济社会发展的支撑能力。全面发展医疗卫生和计划生育事业。增加政府投入，建设覆盖城乡居民的公共卫生服务体系、医疗服务体系、医疗保障体系、药品供应保障体系，为群众提供安全、有效、方便、价廉的医疗卫生服务。完善社区卫生服务网络和功能，提高人口覆盖率。加强农村三级卫生服务网络建设，改扩建10所乡镇卫生院和一批村卫生所。进一步推进新农合工作，提高筹资水平，提高农民参合率，提高患者医药报销比率。发挥好市疾控中心作用，完善疾病防控体系。加强医德医风建设，提高医疗服务质量和水平。坚持计划生育基本国策，稳定低生育水平。深入推进“生殖健康促进计划”和“生育关怀行动”，全面提高出生人口素质和群众健康水平。大力发展文化和体育事业。坚持社会主义先进文化前进方向，建设和谐文化，培育文明风尚，增强文化发展活力。创作具有地域特色和民族风情的文艺精品，打造鹤城文化品牌。挖掘、整理和保护历史文化资源，积极创建国家级历史文化名城。加强文化基础设施建设，加快县级文化馆、图书馆、乡镇文化站和新农村书屋建设，抓好市图书馆建设和工人文化宫改造，丰富群众文化生活。创新文化政策，加快文化产业发展。切实加强广播电视、新闻出版工作，加快电视传输数字化进程，严厉打击盗版和非法出版物及新闻媒体虚假广告等违法经营行为，规范文化市场秩序，促进文化演出和娱乐业健康发展。广泛动员群众参与，挖掘历史文化内涵，把和平节办成具有浓郁地方特色、富有生机活力的市民节日。积极开展全民健身运动，全力做好2008年北京奥运火炬龙江传递第一站的各项工作。努力提高竞技体育水平。举全市之力，把第十一届全国冬运会办成冬运史上最好的一届体育盛会。

　　做好第二次全国经济普查和全国投入产出调查工作，切实关心支持残疾人事业，不断加强妇女儿童工作，进一步做好拥军优抚、防灾救灾、外事侨务、民族宗教、人防、审计、气象、史志、档案、国家安全、民兵预备役等工作。

　　三、加强政府自身建设面对改革、发展、稳定的繁重任务，面对人民群众新的期待，我们必须以高度的政治责任感和使命感，坚持用马克思主义中国化的最新成果武装自己，坚持用时代发展的要求审视自己，坚持用改革创新的精神完善自己，以更加解放的思想观念、更加昂扬的精神状态、更加扎实的工作作风，大力加强政府的自身建设。

　　切实加强学风政风建设。全面加强各级领导干部和机关工作人员的作风建设，大力倡导良好风气，努力建设一支政治坚定、业务精湛、作风过硬、人民满意的公务员队伍。以提高抢抓政策机遇、谋划科学发展、促进社会和谐能力为重点，大兴学习之风，增强全面和正确履行政府职能的能力。以加强机关效能建设为重点，精文简会，大兴调查研究之风，提倡真抓实干，少说多做，力戒官僚主义、形式主义。以加强机关廉政建设为重点，严格执行廉洁自律的各项规定，切实纠正损害群众利益的不正之风，严肃查处违法违纪案件，厉行节约，杜绝铺张浪费，树立良好的政府形象。

　　进一步转变政府职能。理顺政府与企业、市场和社会的关系，在更好地履行经济调节、市场监管职能的同时，切实加强公共服务和社会管理。在公共服务方面，完善服务体系，搞好各级政务大厅建设，加大即时即办服务事项比重。健全行政审批代办制、联审联办制、首办负责制、服务承诺制和限时办结制，提高审批服务“窗口”的办结率。搞好电子政务网络平台建设，逐步完善网上办公、信访、投诉、参与政务、自助服务等功能，提供快捷、直接、多元化、个性化服务。在社会管理方面，完善社会利益诉求和协调机制，积极排查和调处社会矛盾。下放事权，下移管理重心，发挥县（市）区在区域经济发展、城镇建设、公共事务管理等方面的作用。创新政府部门运作方式，优化工作流程，缩短办事周期。

　　提高科学民主决策水平。严格执行重大事项集体决策、专家咨询、社会公示与听证、决策评估等制度，规范决策行为。拓宽人民群众参与渠道，使政府的重大决策特别是利民惠民行动，广泛听取民意，最大限度地保障人民群众对政府决策的知情权、参与权、监督权。加强督办检查，确保各项决策的落实。严格执行决策监督和责任追究制度。改进考核办法，建立以公众评估为主导的绩效考核体系。

　　全面推进依法行政。进一步清理行政权力，明确行政执法权限，减少行政执法层级，完善执法程序，增强政府的公信力和执行力。加强地方法规建设，提高法规制定质量，增强时效性。完善法律顾问制度，将合法性审查的范围从出台政策、法规拓展到部门行政决策，保证政府的职责、权限、行政决定和行政程序有法定依据。清理和调整没有充分体现公平、公正、便民原则的现行政策、法规及行政许可，减少行政审批事项及程序，规范行政执法自由裁量权。自觉接受人大的法律监督、工作监督和政协的民主监督。

　　各位代表，展望未来，鹤城的发展繁荣充满希望。我们的使命崇高而神圣，我们的责任重大而光荣。让我们在市委的领导下，凝聚全市人民的智慧和力量，开拓创新，埋头苦干，努力完成今年的各项工作任务，为实现全面建设小康社会目标而努力奋斗！
